# Evaluation of the Pink Luminous Breast LED-Based Technology Device as a Screening Tool for the Early Detection of Breast Abnormalities

**DOI:** 10.3389/fmed.2021.805182

**Published:** 2022-02-10

**Authors:** Fernando Ocasio-Villa, Luisa Morales-Torres, Norma Velez-Medina, Luis A. Cubano, Juan C. Orengo, Edu B. Suarez Martinez

**Affiliations:** ^1^CEM: Corporación Especial Municipal para el Desarrollo de Investigaciones en Ciencias y Tecnologia de Ponce, Ponce, Puerto Rico; ^2^Public Health Program, Ponce Health Sciences University, Ponce, Puerto Rico; ^3^Research Institute, Ponce Health Sciences University, Ponce, Puerto Rico; ^4^Biology Department, University of Puerto Rico at Ponce, Ponce, Puerto Rico

**Keywords:** breast, mammogram, imaging-based diagnoses, LED-based, early-screening

## Abstract

Breast cancer is the leading cause of sex-specific female cancer deaths in the United States. Detection at earlier stages contributes to decreasing the mortality rate. The mammogram is the “Gold Standard” for breast cancer screening with an estimated sensitivity of 86.9% and a specificity of 88.9%. However, these values are negatively affected by the breast density considered a risk factor for developing breast cancer. Herein, we validate the novel LED-based medical device Pink Luminous Breast (PLB) by comparison with the mammogram using a double blinded approach. The PLB works by emitting a LED red light with a harmless spectrum of 640–800 nanometers. This allows the observation of abnormalities represented by dark or shadow areas. In this study, we evaluated the sensitivity and specificity of the PLB device as a screening tool for the early detection of breast abnormalities. Our results show that the PLB device has a high sensitivity (89.6%) and specificity (96.4%) for detecting breast abnormalities comparable to the adjusted mammogram values: 86.3 and 68.9%, respectively. The percentage of presence of breast density was 78.2% using PLB vs. 72.9% with the mammogram. Even with higher findings of breast density, the PLB is still capable of detecting 9.4% of calcifications compared to 6.2% in mammogram results and the reported findings for cysts, masses, or tumor-like abnormalities was higher using the PLB (6.5%) than the mammogram (5.6%). A 100% of the participants felt comfortable using the device without feeling pain or discomfort during the examination with 100% acceptability. The PLB positive validation shows its potential for routine breast screening at non-clinical settings. The PLB provides a rapid, non-invasive, portable, and easy-to-use tool for breast screening that can complement the home-based breast self-examination technique or the clinical breast examination. In addition, the PLB can be conveniently used for screening breasts with surgical implants. PLB provides an accessible and painless breast cancer screening tool. The PLB use is not intended to replace the mammogram for breast screening but rather to use it as an adjunct or complemental tool as part of more efficient earlier detection strategies contributing to decrease mortality rates.

## Introduction

Breast cancer is the number one female sex-specific cause of cancer death in the United States (1–3). Despite therapeutic advances in the field, the medical and clinical community agree that early detection is the best approach to decrease the mortality rate of this disease (4–7). The objective of breast cancer screening is to detect the disease at a pre-clinical stage in asymptomatic patients to decrease the mortality rate and increase prognostic survival curves, avoiding extreme and expensive interventions with a negative effect in the patient's quality of life. In the clinical practice, the mammogram is the primary imaging-based test and considered as the “Gold Standard” screening test for identifying breast abnormalities, including suspicious lesions for breast cancer. As a public health-oriented screening test, the mammogram is intended to be cost-effective and accessible to the population. Previous studies identified socio-economic status, ethnicity, and health insurance coverage as major variables for the lack of primary screening and follow up visits (7, 8). There are also cultural factors and misinformation within women regarding the importance, significance, and positive outcomes if they undergo routine examinations for detecting breast abnormalities at earlier stages (9). Although the clinical breast examination (CBE) by palpation combined with mammogram increased its sensitivity by 4% (8, 10), it is not approved by most relevant clinical groups guidelines for breast screening (U.S. Preventive Services Task Force (USPSTF) (11), American Academy of Family Physicians (AAFP) (12), ACS). However, the breast self-examination (BSE) is recommended for encouraging breast self-awareness by the American College of Obstetricians and Gynecologists (ACOG), ACS, and the National Comprehensive Cancer Network (NCCN).

Interestingly, the scientific literature suggests that the sensitivity of the mammogram is increased with a complementary secondary image-based test (13, 14).

The scientific literature also expresses some concerns about a decrease in the specificity with Magnetic Resonance Imaging (MRI) (10, 14–17). Furthermore, no other available imaging tools are included in the breast cancer screening guidelines for an average cancer risk person (USPSTF, AAFP, ACS) or for women considered at high risk (USPSTF, AAFP).

There are many studies comparing the sensitivity and specificity of the mammogram with other imaging-based screening techniques such as digital mammogram, ultrasonography, tomosynthesis (3D), MRI, and even positron emission tomography (PET) scan (15, 18–25). Together, these studies concur that current imaging tests, other than the mammogram, despite providing higher sensitivity, require highly regulated settings and other expenses. These include contrasting solutions, prior patient preparations, and specialized personnel among other. These other imaging-based tests provide a clinical added value as supplemental screening after the abnormal mammogram results, for populations classified as having higher risk determined by a breast cancer assessment model, or when another underlying disease is clinically suspected (9, 26).

As the gold-standard screening test, abnormal mammogram observations should generate awareness to promote an informed state of mind about the potential diagnosis and prognosis for the tested person. More importantly, abnormal results are intended to alert physicians about the risks representing these outcomes, such as potential malignancies, and ought to follow the recommended guidelines for patient standard of care on these cases (USPSTF, AAFP, and ACS). Concerningly, Ezratty (24), after a three-years retrospective, observational cohort study of women living in New York, USA, reported that non-Hispanics blacks and Hispanic women are less likely to receive a supplemental imaging-based test follow up order, when compared to non-Hispanic white women. Her group also found that generalist physicians were less likely to appoint follow up visits and order supplemental imaging-based test to these groups, even when presenting suspicious or inconclusive mammogram results, contrasting to specialty physicians (24).

Although the mammogram seems convenient as a breast abnormality screening tool (the benefits super pass the harms), this non-invasive test requires X-Rays exposure, regulated facilities, and trained personnel. It is also well-documented that one of the main reasons discouraging women to perform their scheduled mammogram is the discomfort and pain experience during the testing (27). More importantly, it is questionable if the mammogram is the best screening tool for women with dense or highly dense breast tissue, which is consider a breast cancer risk factor by the cancer assessment models (28, 29). The former brings concerns to the public health and clinical community if there is an underestimation or misdiagnosed (increase false negative results) cases in women from 40 to 74 years of age, especially when those age ranges represent a 50% of dense breast tissue findings and dense breast is considered a risk factor for breast cancer (26, 30–32). For these reasons, new technologies with similar or higher sensitivity and specificity, capable of overcoming expenses, cultural, and religious believes, complains about discomfort, and the potential false negative results on dense breast tissue are highly desirable.

As a potential solution to mammogram limitations other light-based devices have been tested since the 90's but they showed low sensitivity and specificity (33–35). Herein, we introduce a novel LED based and registered FDA class I medical device: The Pink Luminous Breast (PLB). The PLB works by emitting a LED red light with a harmless spectrum of 640–800 nanometers that is safely absorbed by hemoglobin, allowing the transillumination through the breast tissue and observations of abnormalities represented by darker or shadowing areas. In this study, we evaluated the sensitivity and specificity of The PLB device as a screening tool for the early detection of breast abnormalities and cancer suspicious lesions when compared with the mammogram as the gold standard.

## Materials and Methods

**Figure 1:**
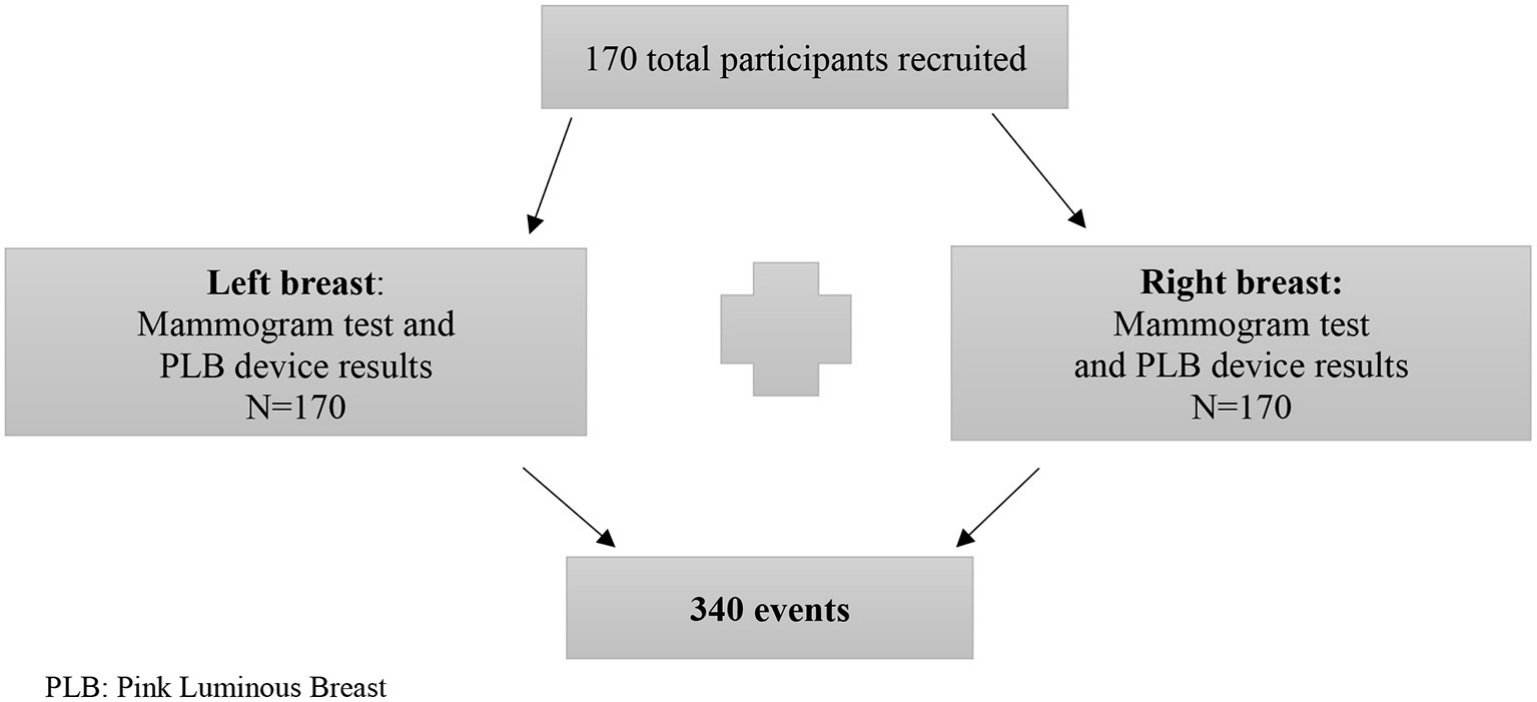
Sample size of the recruited participants and resulting individual events. PLB, Pink Luminous Breast.

A comparison of two diagnostic devices using a double blinded approach comprised of 170 Puerto Rican women, randomly recruited from the general population *via* electronic communications including social media advertising, and flyers displayed at physicians' offices, local clothing stores, and drug stores. For each participant, a mammogram and a PLB image was obtained for each breast, each breast was counted as an independent event for a total of 170 right breast readings and 170 left breast readings for a total of 340 (Figure 1). The study was conducted from June 2020 to December 2020 at the “Centro de Desarrollo de Investigaciones en Ciencias y Tecnología de Ponce, C.D. (CDICTP),” Ponce, Puerto Rico. This study was approved by the Ponce Health Sciences University Institutional Review Board, Ponce, Puerto Rico as protocol #1911024753. In addition, we implemented preventive measurements for COVID-19 as stipulated by the Puerto Rico Governor's Executive Order 2020-018 effective on May 1, 2020.

The inclusion criteria of this study consisted of: Puerto Rican females over 40 years old with printed mammogram results dated from March 2019 to December 2020 as certified by a radiologist. We used the following as exclusion criteria: persons receiving treatment of chemotherapy or radiotherapy, pregnant women, open wounds, infections, lacerations, scratches on the breast or nipple area, breastfeeding, hormonal treatment for fertility, history of autoimmune diseases, and a total or partial mastectomy, unilateral or bilateral.

Upon arrival, the CDICTP Scientific Coordinator (SC) welcomed and escorted the participants to the study office. All participants signed the IRB approved consent form prior to initiating the corresponding assessments. The SC requested from each participant the printed mammogram results to verify if the dates were valid as described at the inclusion criteria section. Then, the SC copied the results and erase the name using a white eraser tape. The SC copied the results without the name, wrote a coding number to the last copy as it appeared in an envelope previously coded, placed the coded copy inside the matching coded envelop, sealed it, and stored it in a locked file cabinet with limited access. This last step is required for confidentiality purposes but in our case, it minimized or avoided *bias* at the time of carrying out the blind analyses. We returned the original and the copy with white eraser tape to the participant. We also instructed the participants not to comment or discuss their mammogram reports with any member of the research staff or other participants waiting for their appointment.

**Figure 2:**
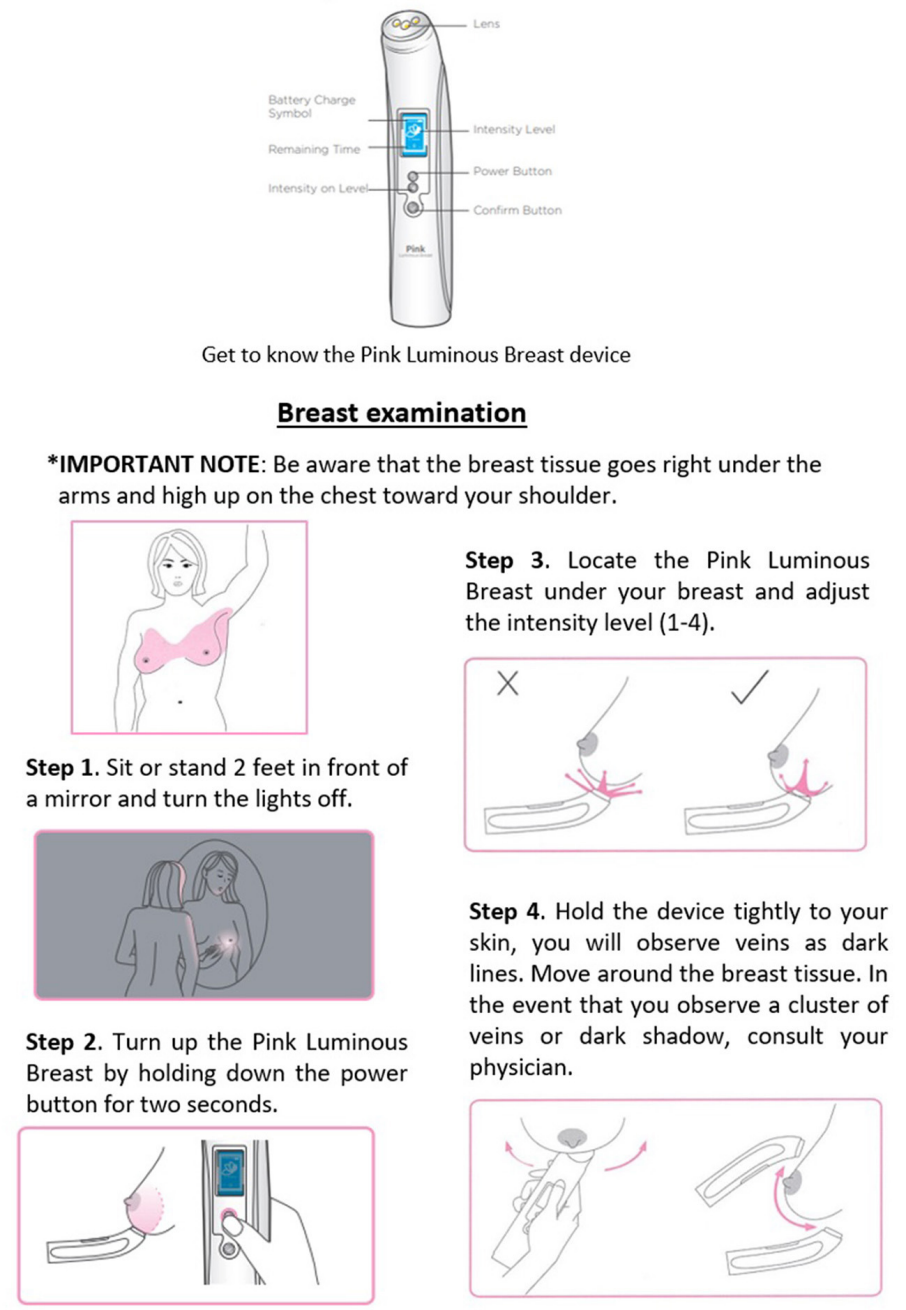
Summary of pink luminous breast instructions.

Then, the participants completed a questionnaire and were escorted by the SC to the examination room. The Study Specialist (SS) presented a 3-min instructional video showing the correct use of the PLB device. Figure 2 represents the steps for using the device. Following the video and prior to starting the examination, the SS described to the participants all the steps to be performed *in-site* and informed the possibility of feeling discomfort such as cold to the touch. The non-invasive PLB device was properly disinfected before and after examination by using an antibacterial solution and 90% ethyl alcohol. The participant stood at 2-feet from a mirror with the room lights turned off. The SS first examined the right breast at three specific areas using the clock as reference for reporting in the following specific order: 6- o'clock, 3-o'clock, and 9-o'clock. The examination areas were documented by a photograph with a digital camera and saved under the corresponding code number, then transferred as a digital file to an external hard drive with password protection in an enclosed locker. We replicated the procedure when examining the left breast. The SS documented additional pertinent notes about the observed breasts such as density of the tissue, presence of fibrotic tissue, evidence of mass or tissue with tumor-related characteristics. Lastly, the participants responded to a survey to evaluate their comfort using the device and the potential for adopting the PLB as a routinely screening tool between an annual mammogram. The SS placed the results in the corresponding previously coded envelop, sealed it, and stored it in a locked file cabinet. The Principal Investigator (PI) started the data analysis by opening the matched coded envelopes (radiologist certified mammogram results and PLB results as reported by the SS) and creating a database using Microsoft Office Excel. Once verified by another colleague, the statistical analyses were performed.

### Sample Size

For this study, we used the sample size estimator from Epidat v 4.2: Estimation for diagnostic test. We used the sensitivity (94.0%) and specificity (89.0%) reported by Bundred et al. (36) and sensitivity (86.9%) and specificity (88.9%) for mammogram examinations based on the Breast Cancer Research Consortium, 2007-2013 (37). For a power of 80% and an alpha of 95%, the estimated minimum sample size was 296 mammograms results. For this reason, a total of 340 events were used in the evaluation of the PLB test performance with each breast considered an independent event (Figure 1).

### Statistical Analysis

The imperfect benchmark test is used when an invasive examination cannot be performed and uses an imperfect reference test whose sensitivity and specificity are known. In this case having a design with a sample of patients who had undergone mammogram and the device under study (PLB), we used a mathematical equation that leads to adequate estimators of the basic indicators of the test under study.


$$\text{S}=\frac{\left(a+b)\beta -b\right)}{\left(a+c\right)-\left(1-\beta \right)N}\text{ E}=\frac{\left(c+d)\alpha -c\right)}{N\alpha -\left(a+c\;\right)}$$

Where α is the sensitivity of the reference test, β is its specificity. The adjusted results of the imperfect benchmark test were used to estimate sensitivity, specificity, positive predictive value, negative predictive value, Youden Index, prevalence and likelihood ratio using Epidat v.3.1. (38).

Descriptive statistics were performed using Stata v 16 for the demographic variable of age and questions related to the acceptability of screening tests for early detection of breast cancer, including acceptability of the device under study (PLB). Additionally, findings related to mammogram results (augmented densities, calcifications and cysts) and PLB positive or negative status were analyzed. Only participants that answered the questions to be analyzed were included, unanswered questions were treated as missing values.

## Results

The mean age at enrollment was 53.3 (SD 9.9) with a range of 40–77 years. At Table 1, we presented the adjusted values by the imperfect reference standard sensitivity for the PLB device 89.6% (95% CI, 84.8–94.3), and specificity of 96.4% (96% CI, 81.3–115.9). Positive predictive value was of 98.8% (95% CI 92.8, 104.4) and negative predictive value 74.4 (95% CI 62.7, 86.1), Youden index was 0.9 (95% CI 0.7, 1.1), after adjustment by the imperfect reference standard (mammogram). Kappa statistic was 0.54, pre-test probability was of 54.7 while the post-test probability was of 98.2%.

**Table 1:** Evaluation of PLB test performance with an imperfect reference standard: Mammogram^*^.

**Parameter**	**Unadjusted results**		Adjusted results^*^	
	**Value**	**95% CI**	**Value**	**95%CI**
Sensitivity (%)	86.3	81.7–90.9	89.6	84.8–94.3
Specificity (%)	68.9	59.6–78.2	96.4	81.3–115.9
Validity index (%)	80.9	76.6–85.2	91.3	85.7–96.7
Predictive value + (%)	86.0	81.3–90.6	98.8	92.8–104.4
Predictive value – (%)	69.5	60.2–78.8	74.4	62.7–86.1
Prevalence (%)	68.8	63.8–73.9	76.2	69.6–82.8
Youden index	0.6	0.5–0.7	0.9	0.7–1.1
Likelihood ratio +	2.8	2.1–3.7	24.9	−145.1–162.1
Likelihood ratio –	0.2	0.1–0.3	0.11	0.06–0.17
Pre-test probability	54.7			
Post-test probability	98.2			
Kappa	0.56			

* *Imperfect reference test (mammogram: sensitivity 86.9%, specificity 88.9%)*.

Table 2 presents the comparison of mammogram with PLB reported findings for breast density, cysts/masses/tumor-like, and calcifications observations. The mammogram identified 248 (72.9%) breast density events, compared to 266 events (78.2%) with the PLB. The percentage of cysts/masses/tumor-like observations was 5.6% (*n* = 19) with the mammogram and 6.5% (*n* = 22) using the PLB examination. Finally, the number of findings for calcifications was a 6.2% (*n* = 21) with the mammogram test and 9.4% (*n* = 32) with the PLB.

**Table 2:** Mammogram findings reports compared to PLB.

	**Mammogram**	**PLB**
**Findings**	**Positive *n* (%)**	**Positive *n* (%)**
Dense breast	248 (72.9)	266 (78.2)
Cyst, mass or tumor-like	19 (5.6)	22 (6.5)
Calcifications	21 (6.2)	32 (9.4)

*PLB, Pink Luminous Breast*.

The data of Table 3 show the acceptability scores for breast cancer screening tools including the PLB. A total of 164 (97%) participants reported being comfortable taking a screening test. All participants (100%) who completed the survey (*n* = 169), answered that the PLB device was easy to use to visualize the breast. None of the participants reported discomfort caused by the device, 100% (*n* = 169). According to our survey, the reasons to avoid a mammogram include: (1) afraid it might hurt 70.4% (*n* = 119); (2) anxiety 20.1% (*n* = 34); and 3) 9.5% (*n* = 16) did not like the mammogram as a screening tool.

**Table 3:** Responses for acceptability of breast cancer screening tools and PLB (*n* = 169).

	***n* (%)**
Age	
Mean (SD)	53.3 (9.9)
Range	40–77
I feel comfortable taking screening tests?	
Yes	164 (97.0)^*^
Is the PLB device easy to use and to visualize my breasts?	
Yes	169 (100.0)^*^
The discomfort felt by the PLB device was:	
None	169 (100.0)^*^
Reasons to avoid a mammogram test	
I don't like it	16 (9.5)
Afraid might hurt	119 (70.4)
Anxiety	34 (20.1)

* *Represents one (1) missing value; PLB, Pink Luminous Breast*.

## Discussion

Breast cancer is still the leading cancer cause of death among women and with an increasing trend of prevalence and mortality rate globally (39). Currently, screening with mammogram is the most effective method to detect the early stage of the disease and decrease mortality (40). However, due to its limitations there exist the need for other breast screening tools that not only exhibit high sensitivity and specificity but also affordability, acceptance by users, and easy to use. Issuing of new safe, accurate, and cost-effective tools for breast screening are essential for appropriate decision making. Accessibility to a sensitive tool for breast self-monitoring will contribute to create awareness, increasing the probabilities of visits to physicians. In developed countries, the PLB could provide a home test screening of the breast that will allow the users to establish a baseline of their breasts and follow changes between scheduled mammograms.

We estimated the screening accuracy of the PLB in detecting breast abnormal or suspicious areas to be 81% with a sensitivity of 89.6%, a specificity of 96.4%, a positive predictive value of 98.8%, and a negative predictive value of 74.4%. Comparable values to those for a mammogram (41) but with the advantage of being portable, rapid, and painless. Importantly, the PLB identified breast density in 78.2% of the reports vs. 72.9% with the mammogram. Even with higher findings of breast density, the PLB is still capable of detecting 9.4% of calcifications compared to 6.2% in mammogram results. Similarly, the percentage of findings for cysts, masses, or tumor-like abnormalities was higher using the PLB (6.5%) than the mammogram (5.6%) (Table 2). As we used the intermediate light setting of the PLB (Level 2), it will be of great importance to further explore if at a higher light setting the PLB is able to detect those lesions missed by the mammogram due to breast density. However, since this was a cross-sectional study, we were not able to follow up on the PLB higher numbers of participants with reported breast abnormalities limiting the comparison of the PLB findings with the final clinical outcomes of the participants.

In this study, the PLB is comparable with the mammogram for its inability to distinguish benign from malignant breast findings, without the outcomes of the pathological examination. To overcome these limitations, in future studies we expect to use a prospective approach following the final diagnosis with both screening tools: the mammogram and the PLB alone and together. Additionally, we will evaluate possible overdiagnosis due to use of the device. We also expect the outcomes of future studies with PLB to lead to further discussions about the ethical limitations for at home screenings as most healthcare systems do not guarantee follow up exams and biopsies to confirm findings from the home screening tests, and referral to treatment facilities (42).

In developed countries, the PLB can be used as an adjunct tool with the mammogram, for improving prognosis by promoting earlier interventions. Breast screening with the PLB device is non-invasive, easy to use, and requiring a short period for examination. Importantly, 100% of the participants reported no discomfort during examination, which increases the probability of its use. Even more, the PLB obtained a 100% of acceptability from the participants (Table 3). In underdeveloped countries, this device may save lives because it will allow breast examinations in places where the mammogram equipment is not affordable or is not available. The PLB is relatively inexpensive, portable, easy to charge, and lasts up to 10 h of use, which will allow for implementation in many regions like other portable electronic devices have been. Under this scenario, based on PLB results, health care personnel can take the corresponding actions to confirm if there is a breast malignancy.

The PLB device has a high sensitivity (89.6%) and specificity (96.4%) for detecting breast abnormalities comparable to the adjusted mammogram values: 86.3 and 68.9%, respectively. Considering this study significant outcomes, we validated the PLB potential to be recommended to be used routinely for breast screening at non-clinical settings. The PLB device provides a rapid, non-invasive, portable, and easy-to-use tool for breast screening that can complement the home-based BSE technique or the CBE. Importantly, the PLB can be conveniently used for screening breast with implants, we plan to study this population in a near future, and male breasts. The use of this device is not intended to replace the mammogram as the gold standard for breast screening but rather to be used as an adjunct or complement tool for earlier detection strategies. At this stage, the main objectives of the PLB is to provide an accessible and painless breast cancer screening tool, to promote awareness about the importance of frequent screening for early detection of breast abnormalities and to decrease this mortal disease.

## Data Availability Statement

The original contributions presented in the study are included in the article. Further inquiries can be directed to the corresponding author.

## Ethics Statement

The studies involving human participants were reviewed and approved by Ponce Health Sciences University Internal Review Board, Ponce Health Sciences University, Ponce, Puerto Rico. The patients/participants provided their written informed consent to participate in this study.

## Author Contributions

FO-V and ES: IRB preparation and submission. FO-V and NV-M: participants recruitment. FO-V: survey preparation, validation, and data base management. NV-M: appointments, performed device tests, prepared tests report notes, and data management. LM-T and JO: statistical analysis and preparation of manuscripts tables. ES: experimental design, data analyses, and submission. LM-T, ES, and JO: literature revision and manuscript preparation. LC: contributed in reviewing the data analyses, editing of the manuscript, introduction, material and methods, results, discussion, revision, and preparation of documents for final re-submission. All authors contributed to the article and approved the submitted version.

## Funding

This work was supported by SILK PRO USA.

## Conflict of Interest

The authors declare that the research was conducted in the absence of any commercial or financial relationships that could be construed as a potential conflict of interest.

## Publisher's Note

All claims expressed in this article are solely those of the authors and do not necessarily represent those of their affiliated organizations, or those of the publisher, the editors and the reviewers. Any product that may be evaluated in this article, or claim that may be made by its manufacturer, is not guaranteed or endorsed by the publisher.
